# The Lessons in the Surgery of War

**Published:** 1862-08

**Authors:** Rufus King Browne

**Affiliations:** Brigade Surgeon U. S. Army, and Chief Medical Officer at Norfolk, Va.


### SELECTED.

### THE LESSONS IN THE SURGERY OF WAR.

BY RUFUS KING BROWNE, M. D.,

BRIGADE SURGEON U. S. ARMY, AND CHIEF MEDICAL OFFICER AT NORFOLK, VA.

About five hundred of those wounded at the battle of "Fair Oaks," who reached Fortress Monroe on transports, were examined by me, and I propose to mention some of the incidents during my attendance on them while at the Naval Hospital, which, particularly, engaged my attention. A large proportion of these, I think a majority, were wounded in the lower part of the body, showing that their enemies had practised the lesson "fire low." The wounds of a considerable proportion, which had laid some hours upon the field, had become a *nisus* for the larvæ of flies, and were occupied by myriads of these at various stages of development, and this was the same where the raw surface was of any greater extent than that pertaining to orifice of entrance or exit, whether it had been made by a missile or by the surgeon's knife in amputation. Nearly all the amputatated cases were at the thigh, and were primary operations which had been very creditably done. Of the five—four of which were primary—which I particularly noted and cared for, three did well and are still doing so. One of the other two had the secondary hæmorrhage, when the femoral was ligated on the eleventh day (so near as I could ascertain) after the amputation, and died the fourteenth day; the other, amputated by myself on the ninth day after the receipt of the wound (a compound a commuted fracture of the femur, extending to the knee-joint), died on the sixth subsequent day, without the supervention of secondary hæmorrhage. I had hoped better, though he was of poorly nourished body and comparatively infirm constitution. Of the three which are doing well, one had secondary hæmorrhage which required ligation of the femoral.

There was one case of amputation of the leg, which confirmed me in my previous view, that where the comminution of the bones of the leg forbids the attempt to save, and requires amputation above the lower third, it had better, as a rule, be done at the knee-joint, and include a thin slice of the femur. In this case, the operation (primary) had been done just below the line of the upper and middle thirds. The thin layer of soft tissue corresponding to the anterior flap, had retracted so far as to bring its apex very nearly into a line with it original base, leaving the end of the protruding bone six or eight lines beyond it. In this case, when it became apparent that the bone must be shortened and covered by the formation of a new flap, I proposed re-amputation at the knee-joint, but by the advice of the surgeon in charge, I operated about two and a half inches above the first. The tissues were found to be morbidly vascular, and though the cut surfaces were not brought together until two hours after, when all oozing had ceased, on the fifth day ligation of the femoral was performed and on the fourth subsequent day the patient died. All these cases were flap operations, as were those I did myself, and all but the latter were infected with maggots. I found no means of removing these except a move at a time by the forceps. Various solutions were tried for the purpose—dilute sol. nitric acid, etc., but were all wholly ineffectual. It occurred to me that quassia, the only bitter tonic which destroys the fly, might destroy the larvæ or disperse them when developed, but I could not obtain this article. Remarkably great in all these amputated cases was the amount of suppuration. In one the drainage of two days amounted to one pint. This, of course, as compared with injury, which consisted merely of the canal formed by the track of the ball. In these latter it constantly seemed to me that there was a very decided difference in the amount of suppuration, whether the wound ran nearly transversely, or nearly longitudinally to the course of the muscles and apeneuroses—there being most in the latter case. But I noticed but little difference in this respect where the wounds respectively involved comminution of the bone.

In none of the amputated cases had the flaps been kept in apposition by union, though in a large number of cases attended to, including the preceeding five, some were progressed in granulation. [I was induced to think that the transportation to a distance from the place of wounding, of amputated cases, or capital cases of the extremities, particularly if such transportation consist of more than one change of conveyance, was not the best surgically. The contrary is undoubtedly the case with sick, or perhaps slightly wounded or injured men. But this opinion is not due to the increased danger during carriage of recurrent hæmorrhage, for this I think unsustained.]

In the dressing of these cases I felt that some simple means should be devised for lifting up the under flap, which constantly tends to weigh away from the upper. All that is required in the case has hitherto been thought to be fully accomplished by the supporting roller about the base of the flaps. But this only keeps the cut surfaces closely pressed. But with this, if the under flap be heavy, it will weigh downwards and backwards, together with the upper which is kept fast to it, so far frequently as plainly to show beneath the latter the whole contour of the cut of the femur. This lifting may be done by passing adhesive straps from a point beyond the base of the flaps, and bringing them forward in one strand to hang upon some elevation at the foot of the bed. The stump up to the point at the base of the flaps where the bevelled form commences, rests upon the opposite cushion. These will, moreover, serve to gently raise the stump from the bed at the time of dressing, avoiding handling, one of the most important of all purposes to the surgeon, and by it also the cushion may be adjusted with the most comfortable nicety.

I am inclined to think that it is preferable to make the posterior stump thinner—not shorter—than usual. The great mass usually left in the lower flap, when pressed up to admit the sutures, forms rounded prominences, not favorable to the object of bringing the surfaces in apposition.

Whoever attended to these wounds with a reflective mind, could not fail to be struck with the consideration that the prevention or arrest of suppuration or a reduction to some attainable minimum, was the paramount consummation. It is not the *amount of pus discharged*, which is so contrary to all the physiological conditions of healthy tissue in the patient for this only indicates the extent of the original morbid irritation which inaugurates the action of the tissues in the suppurative process. This suppuration consists in the enormously rapid multiplication of cells, which immediately and in virtue of the very same conditions which produced them, break down, or as we term it "liquefy."

To reduce or destroy this irritation—to the point where cell multiplication does not exceed normal "granulation"—is the desideratum. *Disinfectants* do not contribute to this end, though they do subserve equally important objects.

The medication in all the preceding cases was done in accordance with my idea of supporting or protecting the *nervous system*—a different thing in my estimation from either supporting or stimulating, i.e. *nourishing* the body. It consisted of quinine and morp. combined, with mild tonics. Where the pain of the cut or lacerated surface was excessive, the latter or pulvis opii was gently scattered upon it with immediate relief. Under the circumstances of such wounds, I am confident that the *assimilation* of however nutritious substances, is nearly or quite precluded by the physiological conditions, and hence nutritious diet is of little avail.—*American Med. Times.*

